# Socio-economic factors associated with delivery assisted by traditional birth attendants in Iraq, 2000

**DOI:** 10.1186/1472-698X-9-7

**Published:** 2009-04-02

**Authors:** Seter Siziya, Adamson S Muula, Emmanuel Rudatsikira

**Affiliations:** 1 Department of Community Medicine, University of Zambia, School of Medicine, Lusaka, Zambia; 2 Division of Community Health, University of Malawi, College of Medicine, Blantyre, Malawi; 3 Division of Epidemiology and Biostatistics, Graduate School of Public Health, San Diego State University, San Diego, California, USA

## Abstract

**Background:**

Traditional birth attendants (TBAs) are likely to deliver lower quality maternity care compared to professional health workers. It is important to characterize women who are assisted by TBAs in order to design interventions specific to such groups. We thus conducted a study to assess if socio-economic status and demographic factors are associated with having childbirth supervised by traditional birth attendants in Iraq.

**Methods:**

Iraqi Multiple Indicator Cluster Survey (MICS) data for 2000 were used. We estimated frequencies and proportions of having been delivered by a traditional birth attendant and other social characteristics. Logistic regression analysis was used to assess the association between having been delivered by a TBA and wealth, area of residence (urban versus rural), parity, maternal education and age.

**Results:**

Altogether 22,980 women participated in the survey, and of these women, 2873 had delivery information and whether they were assisted by traditional birth attendants (TBAs) or not during delivery. About 1 in 5 women (26.9%) had been assisted by TBAs. Compared to women of age 35 years or more, women of age 25–34 years were 22% (AOR = 1.22, 95%CI [1.08, 1.39]) more likely to be assisted by TBAs during delivery. Women who had no formal education were 42% (AOR = 1.42, 95%CI [1.22, 1.65]) more likely to be delivered by TBAs compared to those who had attained secondary or higher level of education. Women in the poorest wealth quintile were 2.52 (AOR = 2.52, 95%CI [2.14, 2.98]) more likely to be delivered by TBAs compared to those in the richest quintile. Compared to women who had 7 or more children, those who had 1 or 2 were 28% (AOR = 0.72, 95%CI [0.59, 0.87]) less likely to be delivered by TBAs.

**Conclusion:**

Findings from this study indicate that having delivery supervised by traditional birth attendants was associated with young maternal age, low education, and being poor. Meanwhile women having 1 or 2 children were less likely to be delivered by TBAs. These factors should be considered in the design of interventions to reduce the rate of deliveries assisted by TBAs in favour of professional midwives, and consequently reduce maternal and neonatal mortality rates and other adverse events.

## Background

Maternal and neonatal morbidity and mortality arising from inadequate health services is an important global health concern. Mortality rates are often higher in low- and mid-income countries where investment in human resources and medical resources are limited [1]. In the past decade, the importance of maternal health has been rekindled following its expected inclusion within the Millennium Development Goals (MDGs); eight in total, the fifth of which is on the reduction of maternal mortality [2,3]. Rasch has argued that skilled birth attendants are a necessity for the reduction of maternal mortality [1].

Darmstadt et al [4] have reported a study in Egypt that while most mothers usually received antenatal care from physicians, traditional birth attendants (TBAs) conducted most deliveries. Anwar et al [5] have reported that low-income families were less likely to have skilled birth attendants at birth, thus raising an equity issue. Using data from Bangladesh, Fonczak et al [6] reported that TBAs with more experience were more likely to use potentially-harmful birthing practices which increased the risk of postpartum morbidity among women with births at home.

Of all causes of maternal deaths in 1999 in Iraq, 46.4% of them were due to bleeding [7]. While the literature is certainly not short of articles that demonstrate the adverse effects of being supervised in delivery by traditional attendants, in many settings of the world however, many women continue to be supervised by this group of health care providers. Women may choose to be delivered by TBAs because of culturally appropriate and respectful care that traditional birth attendants provide [8]. Women may also have TBAs for assistance during delivery because that may be the only option when health facilities and professional trained staff may be less available. In 2002, in Iraq, there were only 7.0 physicians/10,000 population and 8.8 nurses/10,000 population. It is estimated that 50% of all births are home delivered, and TBAs attend 18% of the total deliveries. Neonatal mortality rate/1000 live births and maternal mortality ratio/100000 live births for Iraq have been estimated to be 67.0 and 294, respectively [7].

Using data from the 2000 Multiple Indicator Cluster Survey (MICS) of Iraq, we aimed to assess factors associated with having a delivery supervised by a TBA within the past 12 months. The MICS is coordinated by UNICEF and has as its primary objectives the following: to provide up-to-date information for assessing the situation of children and women at the end of the decade and for looking forward to the next decade; "to furnish data needed for monitoring progress toward goals established in 1990 at the World Summit for Children and as a basis for future action and; to contribute to the improvement of data and monitoring systems a country and to strengthen technical expertise in the design, implementation, and analysis of such systems" [9].

## Methods

### Survey design

A description of the MICS methodology is reported elsewhere [9,10]. However, in brief, the sample was drawn using a 3-stage stratified sampling method. During the first stage of sampling, *mahallas (villages) *were selected with probability proportional to population size in each sub-district (*Qada'a*). In the second stage, *mahallas *were divided into compartments with population of about 500 people, and one or more compartments were then selected from each *mahalla*. Further, each compartment was divided into *Majals *(blocks) of 25–30 households in urban area and 20–25 households in rural areas. One *Majal *was then selected by simple random sampling from each compartment. Finally, a cluster of 10 households was selected from each *Majal *using a systematic random sampling technique. All18 governorates of the country were represented in the sample.

### Questionnaire administration

A standardized MICS questionnaire provided by UNICEF was translated into Arabic by the Middle East and North Africa Regional Office of UNICEF (MENARO), with some revisions and adaptations. The revised questionnaire was pretested in August 2000.

Of the 13,220 women who had delivered a child 13,114 (99.2%) were eventually interviewed as women who had delivered a child within the last 12 month.

### Ethical consideration

The Iraqi MICS survey of 2000 was conducted by the government with technical support from UNICEF. We obtained de-identified data from UNICEF which we analysed within the current secondary study. Our analysis of de-identified secondary data was exempted from full institutional review board.

### Data analysis

We conducted weighted analysis using SPSS version 11.5 (Statistical Package for Social Sciences, Chicago, Illinois, United States). We obtained frequencies and proportions to describe the sample with regard to the outcome of interest (i.e. having been supervised by a TBA in delivery occurring within the last 12 months) and other socio-demographic characteristics.

The outcome variable was categorized into being assisted during delivery by TBAs and being assisted by health professional (doctor, nurse/midwife, and auxiliary midwife) or relative/friend. Marital status was categorized as: currently married/in union and not currently married. The not currently married included women who were widowed, divorced, separated or had never been married. Age was taken as completed years at the time of the survey. Wealth was measured by using household assets (such as radio, bicycle, car, television, type of roofing, and floor) rather than income as has been done in other community surveys such as the Demographic and Health Survey [11-13]. Each asset was assigned a weighting value, using principal component analysis as described by the World Bank and ORC Macro. A household was assigned a standardized score for each owned asset, and these scores were summed and households ranked into five wealth quintiles.

We conducted bivariate and multivariate logistic regression methods to assess associations between predictor variables and the outcome. The unadjusted odds ratio (OR), and adjusted odds ratio (AOR) together with their 95% confidence intervals (CI) are reported. For the purpose of our study, traditional birth attendants were not considered skilled or professional birth attendants.

## Results

Altogether 22,980 women participated in the survey, and of these women, 2873 had delivery information and whether they were assisted by traditional birth attendants (TBAs) or not during delivery. About half of the women were of age 25–34 years (49.1%), and almost all of them were married (99.3%). The majority of the respondents had not gone further than primary level of education (74.1%). Most of the respondents resided in urban areas (62.8%). About 1 in 5 women (26.9%) were assisted during delivery by TBAs. Further description of the sample with regard to key socio-demographic variables is shown in Table 1.

**Table 1:** Socio-demographic description of on women who delivered within the last 12 months in the Iraq MICS of 2000

	Total
Factor	n (%)
Age (years)	
<25	861 (31.1)
25–34	1399 (49.1)
35+	613 (19.8)
	
Currently married	
Yes	2851 (99.3)
No	22 (0.7)
	
Maternal education level	
None	918 (27.9)
Primary	276 (46.2)
	
secondary or higher	640 (25.9)
Area of residence	
Urban	1481 (62.8)
Rural	1388 (37.2)
	
Wealth index (Quintiles)	
Poorest	946 (25.0)
Second	546 (19.7)
Middle	574 (20.8)
Fourth	416 (21.1)
Richest	387 (13.4)
	
Number of children ever born	
1 or 2	877 (31.4)
3 or 4	815 (29.9)
5 or 6	568 (19.0)
7 or more	611 (19.7)
	
Delivery assisted by Traditional birth attendant	
No	2070 (73.1)
Yes	803 (26.9)

Note: numbers are not adding up because of non responses to some variables

Table 2 shows factors associated with traditional birth attendant assisted-delivery that were considered in the analysis. Only age and marital status were not significantly associated with TBA assisted-delivery at bivariate analyses. At multivariate analysis, marital status and area of residence were not significantly associated with TBA assistance during delivery. Compared to women of age 35 years or more, women of age 25–34 years were 22% (AOR = 1.22, 95%CI [1.08, 1.39]) more likely be assisted by TBAs during delivery. Women who had no formal education were 42% (AOR = 1.42, 95%CI [1.22, 1.65]) more likely to be delivered by TBAs compared to those who had attained secondary or higher level of education. Women in the poorest and middle wealth quintiles were 2.53 (AOR = 2.52, 95%CI [2.14, 2.98]) more likely, and 25% (AOR = 0.75, 95%CI [0.62, 0.92]) less likely, respectively, to be delivered by TBAs compared to those in the richest quintile. Compared to women who had 7 or more children, those who had 1 or 2 were 28% (AOR = 0.72, 95%CI [0.59, 0.87]) less likely to be delivered by TBAs.

**Table 2:** Unadjusted odds ratios (OR) and adjusted odds ratios (AOR) and 95% confidence interval (CI) of the association of socio-demographic variables and delivery assisted by traditional birth attendant in Iraq, 2000

Characteristics	OR (95%CI)	AOR (95%CI)
Age (years)		
<25	0.83 (0.64, 1.06)	1.03 (0.86, 1.24)
25–34	1.19 (0.95, 1.49)	1.22 (1.08, 1.39)
35+	1	1
		
Currently married		
Yes	0.73 (0.46, 1.17)	-
No	1	
		
Maternal education level		
None	1.86 (1.64, 2.12)	1.42 (1.22, 1.65)
Primary	1.08 (0.96, 1.22)	1.05 (0.92, 1.20)
Secondary or higher	1	1
		
Area of residence		
Urban	0.66 (0.60, 0.72)	-
Rural	1	
		
Wealth index (Quintiles)		
Poorest	2.90 (2.49, 3.39)	2.52 (2.14, 2.98)
Second	1.27 (1.06, 1.52)	1.14 (0.95, 1.37)
Middle	0.74 (0.61, 0.89)	0.75 (0.62, 0.92)
Fourth	0.79 (0.65, 0.96)	0.85 (0.69, 1.03)
Richest	1	1
		
Number of children ever born		
1 or 2	0.66 (0.57, 0.76)	0.72 (0.59, 0.87)
3 or 4	1.01 (0.88, 1.16)	1.04 (0.89, 1.21)
5 or 6	1.11 (0.95, 1.30)	1.07 (0.90, 1.28)
7 or more	1	1

## Discussion

In a study of Iraq women in which data on delivery supervision at the most recent delivery was available for 2,873 women, 26.9% reported that they had been delivered by TBAs. In multivariable analysis we found that younger women in the age group 25 to 34 years were 22% more likely to report having been delivered by TBAs compared to women 35 years and older. We also found that higher parity, middle-income category, low education and rural residence were positively associated with delivery by traditional birth attendant.

The finding that women in the middle wealth category were less likely to be delivered by TBAs compared to women in the richest category may be a spurious finding because the odds of being delivered by TBAs were not significantly different between women in the second and fourth categories on one hand and women in the richest category on the other. Women in the lowest wealth quintile were more likely to deliver under TBA supervision than women in the richest wealth category. This finding is similar to what has been published elsewhere where data have shown that women in poor households are less likely to receive professional medical care [14,15].

Access to health care facilities may be limited as a result of transport and hospital fees [16]. Lawoyin [17] has reported high neonatal adverse outcomes among deliveries occurring out of health care facilities. While facility deliveries are preferred wherever possible, Leigh et al [18] have reported on poor maternal outcomes in Malawi within health facilities, largely because of low quality of care. Furthermore, supervised midwifery deliveries at home must be differentiated from low-skilled traditional birth attendant supervision. Just because a delivery occurs at home should not necessarily imply it is of low quality. In an environment where transport and communication facilities are available, delay can be minimised, safe home deliveries can be provided, while expecting that transfer to a facility with capabilities to provide higher level care is possible [19,20].

We also found that parity i.e. the number of children a woman has ever delivered was associated with TBA supervisions. Women who may have previous non-eventful deliveries may be complacent and expect that TBA assisted deliveries are safe. Primiparas may have been less likely to be delivered by TBAs because they may not have been sure of their pregnancy outcome, and would have liked to be attended by professional skilled persons during delivery.

Women with low education were more likely to be delivered under the supervision of TBAs. The higher likelihood of TBA deliveries by low education women may also be associated with limited appreciation by women with limited education to understand the need for professional supervision during delivery. Our findings is similar to that reported by Tann et al [21] that skilled birth attendants remain difficult to be accessed by less educated women.

While this study has obvious strength such as the design being tailored to produce nationally representative data and use of a standard MICS questionnaire, there are a number of limitations worth considering. In comparing these results with similar studies in which rural versus urban residence status was assessed, it is important to remember that the definitions of urban versus rural may differ from country to country [22-24]. In the Iraq MICS, an urban area was defined as any administrative setup lying within the municipality boards; areas other than these were considered rural. Furthermore, data were collected via self-reports.

To the extent that study participants intentionally or inadvertently misreported, our findings may be biased. Finally, although the data collected were cross sectional in nature, and we are not able to ascertain causation between any of the variables and the outcome [25] i.e. having been delivered by a traditional birth attendant, the current findings are consistent with those of the previous studies. These factors should be considered in designing interventions to reduce the rate of TBA assisted deliveries, and hence reduce neonatal and maternal mortality rates, rather than generating hypotheses and considering similar future research using study designs that are higher than cross sectional studies. Missing information in the Iraq 2000 MICS data is of concern, and our findings may be biased to the extent that non-respondents differed from those that responded to the questionnaire items we considered in our analysis.

## Conclusion

Findings from this study indicate that having delivery supervised by traditional birth attendants was associated with young adult age, low education, and being poor. Meanwhile, women having 1 or 2 children were less likely to be delivered by TBAs. These factors should be considered in the design of interventions aimed to promote professional delivery with the consequent aim of reducing the proportion of deliveries assisted by TBAs. This may result in the reduction of maternal and neonatal mortality rates.

## Competing interests

The authors declare that they have no competing interests.

## Authors' contributions

SS conducted the data analysis, participated in the interpretations of the results and drafting of the manuscript. ASM participated in the interpretations of the results and drafting of the manuscript. ER participated in the interpretations of the results and drafting of the manuscript. All authors read and approved the final manuscript.

## Pre-publication history

The pre-publication history for this paper can be accessed here:

http://www.biomedcentral.com/1472-698X/9/7/prepub
